# Knowledge, affirmativeness, and experience as factors associated with self-efficacy in LGBTQ+-affirmative services for individuals with IDD

**DOI:** 10.3389/fpsyt.2026.1817785

**Published:** 2026-07-23

**Authors:** Ivanka Simić Stanojević, Jennifer A. Piatt, Alison Greene, Catherine Sherwood-Laughlin, William Ramos

**Affiliations:** 1 School of Public Health-Bloomington, Indiana University, Bloomington, IN, United States; 2 School of Health and Human Sciences-Indianapolis, Indiana University, Indianapolis, IN, United States

**Keywords:** cultural competence, direct support professionals, disability services, intellectual and developmental disability (IDD), intersectionality, LGBTQ+, LGBTQ+ affirmative practice, self-efficacy

## Abstract

**Introduction:**

Lesbian, gay, bisexual, transgender, and queer+ (LGBTQ+) individuals with intellectual and developmental disabilities (IDD) experience compounded discrimination stemming from the intersection of disability and sexual and gender minority identities. Direct support and service-providing professionals (DSPs) play a critical role in shaping inclusive service environments; however, perceived lack of competence may contribute to the avoidance of sexual identity-related topics and unmet support needs. Despite the importance of affirmative practice within disability services, limited research has examined factors associated with DSPs’ self-efficacy in providing LGBTQ+-affirmative services and care to individuals with IDD.

**Methods:**

Guided by Bandura’s self-efficacy theory, this cross-sectional study examined self-efficacy among 114 DSPs working with individuals with IDD, including special educators, teachers, recreational and occupational therapists, nurses, social workers, psychologists, and behavioral professionals and identified factors associated with variability in perceived competence. Participants completed adapted versions of the Lesbian, Gay, and Bisexual Affirmative Counseling Self-Efficacy Inventory and the Lesbian, Gay, and Bisexual Knowledge and Attitudes Scale. Correlation and hierarchical multiple regression analyses were conducted to examine associations between LGBTQ+-related knowledge, attitudes, experience, and self-efficacy.

**Results:**

LGBTQ+-specific knowledge demonstrated the strongest and most consistent associations with self-efficacy across domains. In multivariable analyses, knowledge emerged as the strongest independent correlate of self-efficacy, followed by prior experience working with LGBTQ+ individuals with IDD. General training specific to working with individuals with IDD alone was not associated with higher self-efficacy. Affirming attitudes were positively associated with self-efficacy, but this association weakened when knowledge and experience were included.

**Discussion:**

These findings highlight the domain-specific nature of self-efficacy in LGBTQ+-affirmative practice and stress the importance of targeted, intersectionally-informed training that integrates knowledge development with experiential learning. Strengthening DSPs’ perceived competence may contribute to more inclusive service-providing settings and improved well-being for LGBTQ+ individuals with IDD.

## Introduction

Individuals with intellectual and developmental disabilities (IDD) who identify as lesbian, gay, bisexual, transgender, or queer+ (LGBTQ+) experience compounded discrimination resulting from the intersection of disability and sexual or gender minority identities (1–5). Diagnosed before the age of 22, IDD are characterized by limitations in intellectual functioning and adaptive behavior that affect every day social and practical skills (6). Harmful misconceptions about the sexuality of individuals with IDD include being perceived as either asexual or hypersexual—a false and harmful dichotomy that negatively affects autonomy and leads to increased social exclusion (7–11). For LGBTQ+ individuals with IDD, these ableist assumptions intersect with heteronormativity and cisnormativity, contributing to marginalization and limiting access to affirming services and supports, placing them at a higher risk of sexual victimization and adverse health outcomes (2, 4, 11–14).

Direct support and service-providing professionals (DSPs) hold a critical role in shaping the quality, inclusivity, and responsiveness of services for individuals with IDD. Providing inclusive services, resources, and support across service settings has the potential to enhance well-being and foster more fulfilling lives for LGBTQ+ individuals with IDD. Through everyday interactions, policy implementation, and advocacy, DSPs influence whether LGBTQ+ identities are affirmed or further marginalized within service-providing settings. However, research indicates that although some professionals consider sexual health as part of their professional practice (15–18), many report feeling underprepared, uncomfortable, or lacking confidence when addressing sexuality topics with individuals with IDD (19–22). These challenges stress the need for structured, identity-affirming professional development that prepares DSPs to provide equitable and culturally-responsive services and care.

The experiences of LGBTQ+ individuals with IDD are best understood through an intersectional framework. Intersectionality, introduced by Kimberlé Crenshaw (23), posits that social identities such as disability, sexual orientation, gender identity, race, and class do not operate independently but interact within broader systems of power. Rather than representing additive forms of disadvantage, these identities intersect within structures shaped by ableism, heteronormativity, and cisnormativity, producing unique patterns of marginalization and access to resources. Intersectionality has been widely applied across public health and social sciences (24–28) to examine how structural inequities shape lived experience and well-being among multiply marginalized populations. Applying this lens to LGBTQ+ individuals with IDD highlights the need for service models that explicitly address intersecting identities rather than relying on disability or LGBTQ+ frameworks alone.

LGBTQ+-affirmative practice has emerged as an approach designed to support individuals navigating stigmatizing social environments (29–33). LGBTQ+ affirmative therapy integrates practitioner knowledge of LGBTQ+ developmental and cultural experiences, self-awareness of biases, and the translation of this awareness into affirming professional behaviors (34). Although initially conceptualized within psychotherapy, affirmative principles extend across service contexts and are highly relevant for DSPs working with individuals with IDD. For LGBTQ+ individuals with IDD, affirmative practice requires attention to how disability and sexual and gender minority identities intersect to shape autonomy, consent, vulnerability, and social inclusion. Developing training structured within an intersectionality framework is therefore essential to ensure equitable and LGBTQ+-affirmative service delivery for LGBTQ+ individuals with IDD.

However, knowledge of LGBTQ+-affirmative principles alone does not guarantee their effective implementation. Bandura’s self-efficacy theory provides a foundational framework for understanding how beliefs about one’s capabilities influence professional behavior (35, 36). Self-efficacy influences activity selection, effort, persistence, and emotional responses in challenging situations (37). Professionals with lower perceived self-efficacy may avoid conversations related to sexual orientation and/or gender identity, particularly when working with populations historically desexualized or stigmatized, such as individuals with IDD. In contrast, stronger efficacy beliefs are associated with greater engagement, persistence, and adaptive professional behavior (37). Thus, self-efficacy—alongside adequate knowledge and skills—is central to initiating and sustaining the avoided challenging or uncomfortable professional activities and delivering LGBTQ+-affirming services to individuals with IDD.

Self-efficacy theory has been widely applied across service-providing professions, including education, psychology, nursing, and healthcare (38–44). Research consistently demonstrates that training and educational interventions can significantly improve professionals’ self-efficacy, which in turn is associated with enhanced work engagement, performance, and quality of care (38, 45–49). The adoption of evidence-based practices relies heavily on providers’ confidence in delivering them, making self-efficacy a critical component of successful implementation (40).

Self-efficacy has practical implications for service quality because professionals who believe they can perform specific tasks are more likely to engage persistently, use effective strategies, and respond adaptively in challenging situations. Prior research across education and healthcare demonstrates that higher professional self-efficacy is associated with improved practice behaviors and outcomes (50–52). For example, teachers with higher self-efficacy are more likely to use learner-centered and constructive instructional approaches, stronger classroom management strategies, with their showing improved academic achievement (51, 52). Similarly, among nurses, higher professional self-efficacy has been associated with safer and better service provision and reduced missed nursing care, suggesting that confidence in one’s professional capabilities can translate into more consistent and higher-quality care delivery (50). Applied to LGBTQ+-affirmative practice with individuals with IDD, these findings suggest that DSPs with higher self-efficacy may be more likely to initiate identity-related conversations, use affirming language, advocate for inclusive supports, and provide responsive services rather than avoiding potentially uncomfortable topics.

Studies in educational and healthcare settings further demonstrate that domain-specific knowledge and attitudes are associated with self-efficacy. For example, teachers’ self-efficacy in working with autistic students has been positively associated with knowledge and attitudes toward that population (39). Similarly, research among healthcare providers shows that self-efficacy is central to effective delivery of health education and professional competence (44). These findings suggest that targeted knowledge acquisition and experiential training are key mechanisms underlying the development of professional self-efficacy.

Bandura (35) identifies four primary sources of self-efficacy: mastery experiences, vicarious experiences, verbal persuasion, and physiological and emotional states. In the context of LGBTQ+-affirmative practice with individuals with IDD, mastery experiences may involve successfully facilitating LGBTQ+-affirming interactions; vicarious experiences may occur through observing successful modeling of affirming language and inclusive practices; verbal persuasion may involve a supportive supervision and training with positive feedback and encouragement; and emotional regulation may involve managing personal discomfort or bias when addressing sexual identity-related issues. These sources indicate that self-efficacy in LGBTQ+-affirmative practice is domain-specific and determined by targeted knowledge, experiential exposure, and supportive professional and learning environments (53–57).

Given the intersecting marginalization experienced by LGBTQ+ individuals with IDD and the central role of DSPs in shaping inclusive service environments, understanding factors associated with DSPs’ self-efficacy in LGBTQ+-affirmative practice is essential. Guided by intersectionality theory and Bandura’s self-efficacy framework, this study examines DSPs’ perceived self-efficacy in providing LGBTQ+-affirmative services to individuals with IDD and identifies factors associated with variability in self-efficacy. Understanding these associations may inform the development of training initiatives tailored to improve self-efficacy and LGBTQ+-affirmative service provision for individuals with IDD across diverse practice settings.

## Methods

This study employed a cross-sectional survey design administered through the Qualtrics^®^ online survey platform. Institutional Review Board (IRB) approval was obtained from the first author’s institution prior to the initiation of all study procedures. Participants were recruited by the first author using multiple methods, including outreach to professional listservs; professional, student, and scientific associations and organizations; and public provider directories. Recruitment information was also posted in private Facebook groups after the first author requested group access and obtained moderator permission to share the study invitation. Snowball sampling occurred through professional connections with DSPs, who shared the recruitment message with eligible colleagues. Recruitment materials included a brief description of the study’s purpose and eligibility criteria, along with a link directing potential participants to the online survey. Upon clicking the survey link, participants were first presented with a study information sheet outlining the purpose of the research, procedures, potential risks and benefits, voluntary nature of participation, and confidentiality protections. Participants were informed that any identifying information they provided would be de-identified. Participants who did not complete items from The Lesbian, Gay, and Bisexual Affirmative Counseling Self-Efficacy Inventory (LGB-CSI) and The Lesbian, Gay, and Bisexual Knowledge and Attitudes Scale (LGB-KAS) were discarded. Participants were then asked to provide informed consent electronically. Only individuals who indicated their consent were permitted to proceed to the survey. Participants who declined consent were excluded from the study without providing any data. Following consent, participants completed an online questionnaire that included demographic items and standardized measures assessing self-efficacy, knowledge, and attitudes regarding providing LGBTQ+-affirmative services to individuals with IDD. Survey completion required approximately 15 minutes. Participants were asked to provide their email addresses if they wished to be entered into a drawing for one of 20 randomly selected $20 electronic gift cards. Upon completing the survey, participants were invited to provide an email address for the purpose of distributing the incentive. Random selection of incentive recipients was conducted using the Microsoft Excel randomizer tool. An *a priori* sample size estimate indicated that approximately 100 participants would be sufficient to detect a medium effect size with acceptable statistical power. Given the absence of prior studies employing comparable designs and the unknown size of the eligible population, this estimate was informed by statistical considerations and feasibility constraints. Sample size reflected feasibility considerations and statistical power estimates for detecting medium effects.

### Participants

A total of 126 participants were recruited for this study. Inclusion criteria required participants to (a) be a DSP, including but not limited to special educators, teachers, recreational therapists, occupational therapists, nurses, social workers, psychologists, and behavioral professionals; (b) have a minimum of three months of professional experience within the past five years or currently be engaged in professional activities with individuals with intellectual and developmental disabilities (IDD); (c) be at least 18 years of age; and, (d) reside in the United States. Participants were required to have at least 3 months of professional experience working with individuals with IDD. This short time frame allowed for the inclusion of early-career professionals, yet ensured sufficient professional exposure to support meaningful reflection on practice. Additionally, eligibility was limited to those with experience within the past five years to enhance the relevance and accuracy of responses, reflecting current service contexts, practices, and standards, while also reducing potential recall bias.

### Measures

#### Participant characteristics

We asked participants to respond to 13 demographic questions about their age, race and ethnicity, gender identity, sexual orientation, education, occupation, geographic location, and religious service attendance.

#### Professional training

We asked participants two Yes/No questions related to professional training: (1) “Have you undergone any training or received professional education specifically for working with individuals with intellectual and developmental disabilities (IDD)?”; and (2) “Have you undergone any training or received professional education specifically for working with lesbian, gay, bisexual, transgender, and queer+ (LGBTQ+) individuals?”

#### Professional experience

We asked participants two questions related to their professional experience: (1) “How extensive is your experience in working with individuals with IDD?” The response options were: Less than 6 months; Between 6 months and 2 years; Between 2 and 5 years; Between 5 and 10 years; More than 10 years; and (2) “To your knowledge, have you ever worked with a LGBTQ+ client/patient/student with IDD?” (Yes, No, I do not know).

#### The lesbian, gay, and bisexual affirmative counseling self-efficacy inventory

The LGB-CSI (58) was adapted to assess DSPs’ self-efficacy in providing LGBTQ+-affirmative services to individuals with IDD. Adaptations included updating terminology from “LGB” to “LGBTQ+” and modifying item wording to refer to “LGBTQ+ clients, patients, or students with IDD”. For example, “How confident am I in my ability to … Directly apply sexual orientation/identity development theory in my clinical interventions with lesbian, gay, and bisexual (LGB) clients” was adapted to “How confident am I in my ability to … Directly apply sexual orientation/identity development theory in my practice with lesbian, gay, and bisexual (LGBTQ+) clients with intellectual and developmental disabilities (IDD).” These modifications were intended to align the measure with the service context examined in this study. However, because LGBTQ+-affirmative practice with individuals with IDD may involve additional considerations related to communication, autonomy, supported decision-making, dependency, and vulnerability to abuse, the adapted measure should be interpreted as assessing perceived self-efficacy within this specific professional context.

The 32-item instrument measures confidence across five domains of affirmative practice: advocacy skills, application of knowledge, awareness, assessment, and relational skills. Although originally developed for mental health professionals, the LGB-CSI has been modified and used across diverse service-providing contexts (59–62). Items were rated on a 6-point Likert scale ranging from 1 (Not at all confident) to 6 (Extremely confident), with higher scores indicating greater perceived self-efficacy. The LGB-CSI and its subscales have demonstrated strong internal consistency (α >.70), supported by evidence of content, construct, and convergent validity, as well as discriminant validity, as indicated by minimal associations with social desirability and impression management (58, 63). Although test-retest reliability has been reported as modest over a two-week interval, the measure is appropriate for cross-sectional research.

#### The lesbian, gay, and bisexual knowledge and attitudes scale

The LGB-KAS (64) was used to assess DSPs’ knowledge and attitudes toward LGBTQ+ individuals and communities. For this study, we updated the terminology from “LGB” to “LGBTQ+” to reflect contemporary and more inclusive language. For example, “I have close friends who are LGB” was adapted to “I have close friends who are LGBTQ +.” The LGB-KAS was not adapted to assess attitudes toward LGBTQ+ individuals with IDD specifically; rather, it was used to assess LGBTQ+-related knowledge and attitudes more broadly. Similar terminology updates have been used in prior research applying LGBTQ+-related knowledge and attitude measures in contemporary professional and educational contexts (65, 66).

The 28-item scale assesses five factors: knowledge, internalized affirmativeness, hate, religious conflict, and civil rights attitudes. The Knowledge subscale assesses foundational knowledge of LGBTQ+ communities, including awareness of LGBTQ+ organizations, key historical movements, and community symbols (e.g., “I am knowledgeable about the significance of the Stonewall Riot to the Gay Liberation Movement”). The Internalized Affirmativeness subscale measures participants’ proactive engagement with LGBTQ+ advocacy and comfort with same-sex attraction (e.g., “I would display a symbol of gay pride (pink triangle, rainbow, etc.”). The Hate subscale assesses negative emotional and attitudinal responses toward LGBTQ+ individuals, including avoidance, hostility, and endorsement of violence (e.g., “It is important to me to avoid LGBTQ+ individuals”). The Religious Conflict subscale measures tension between religious beliefs and acceptance of LGBTQ+ individuals, including negative religious attitudes toward LGBTQ+ identities (e.g., “I keep my religious views to myself in order to accept LGBTQ+ people”). The Civil Rights Attitudes subscale assesses support for LGBTQ+ civil rights, including marriage equality, parenting rights, healthcare access, and recognition of same-sex partners in healthcare decision-making contexts (e.g., “Hospitals should acknowledge same-sex partners equally to any other next of kin”). Items were rated on a 6-point Likert scale ranging from 1 (Very uncharacteristic of me or my views) to 6 (Very characteristic of me or my views), with higher scores indicating greater knowledge and more affirming attitudes. The LGB-KAS has demonstrated adequate internal consistency (α >.70), strong test–retest reliability, and evidence of construct, convergent, and discriminant validity (64, 67).

### Data analysis

Descriptive statistics were calculated to summarize participant demographic characteristics. We used the data obtained from the LGB-CSI and LGB-KAS to examine relationships between DSPs’ self-efficacy and their knowledge and attitudes toward LGBTQ+ populations. Correlational analysis was conducted using Kendall’s tau (τ), a nonparametric statistic selected for its robustness to outliers and suitability for skewed or non-normally distributed data. We performed the multiple linear regression analysis to examine factors associated with DSPs’ self-efficacy in providing LGBTQ+-affirmative services to individuals with IDD. Predictor variables included LGBTQ+-related knowledge and attitudes, prior experience working with LGBTQ+ individuals with IDD, training, and relevant demographic characteristics (e.g., profession, age, and LGBTQ+ identity). For regression analyses, LGBTQ+ identity and IDD- and LGBTQ+-specific training were coded dichotomously for group comparisons. Continuous predictor variables (LGB-KAS Knowledge and Internalized Affirmativeness) were entered in their original scale metrics. We evaluated regression models to identify significant predictors and the model that best explained variance in self-efficacy. All statistical tests were conducted using a significance level of *p* = .05. Analyses were performed using IBM SPSS Statistics, Version 30.

Because we adapted the LGB-CSI and LGB-KAS for use in the present study, we examined internal consistency for the total scales and subscales in this sample. We calculated Cronbach’s alpha for the LGB-CSI total scale and its five subscales—Knowledge Application, Awareness, Advocacy Skills, Assessment, and Relationship—as well as for the LGB-KAS total scale and its five subscales—Knowledge, Internalized Affirmativeness, Hate, Religious Conflict, and Civil Rights Attitudes. Item-deletion diagnostics were reviewed to assess whether removal of individual items would meaningfully improve or reduce internal consistency. We compared reliability estimates descriptively with previously published psychometric values for the original instruments to assess whether the adapted versions demonstrated comparable internal consistency. These analyses provide preliminary reliability evidence for the adapted measures in the present sample but do not constitute a full validation of the instruments.

## Results

### Internal consistency of adapted measures

Internal consistency analyses indicated that the adapted LGB-CSI demonstrated excellent overall internal consistency, with subscale reliabilities ranging from good to excellent in the present sample (α = 0.88–0.97). The total LGB-CSI score had a raw Cronbach’s α of 0.97. Reliability estimates for the Knowledge Application, Awareness, Advocacy Skills, Assessment, and Relationship subscales were 0.97, 0.88, 0.95, 0.92, and 0.90, respectively. These raw alpha values were generally consistent with previously reported reliability estimates for the original instrument (58, 63).

The LGB-KAS demonstrated strong internal consistency for the total scale, with subscale reliabilities ranging from marginal to good (α = 0.65–0.88). The total LGB-KAS score had a raw Cronbach’s α of 0.93. Reliability estimates for the Knowledge, Internalized Affirmativeness, Hate, Religious Conflict, and Civil Rights Attitudes subscales were 0.80, 0.88, 0.65, 0.87, and 0.83, respectively. For the Hate subscale, one item was omitted from the subscale reliability calculation because all participants provided the same response, resulting in no item-level variance and no contribution to internal consistency. Although the raw alpha for the Hate subscale (α = 0.65) was lower than that of the other subscales, the standardized alpha and McDonald’s omega were both 0.73, suggesting adequate internal consistency when accounting for item scaling and covariance structure. Overall, these values were broadly comparable with previously reported psychometric values for the original scale (64).

### Participants

Sociodemographic characteristics are presented in **Table 1**. A total of 114 participants were included in the final analyses after applying exclusion criteria for incomplete scale data. The majority were between 18 and 44 years old (78.9%). Most participants identified as white (*n* = 104, 91.2%). In terms of gender, the sample consisted predominantly women (*n* = 96, 84.2%), followed by men (*n* = 13, 11.4%) and gender nonbinary individuals (*n* = 4, 3.5%). Most participants identified as heterosexual (*n* = 92, 80.7%). Participants resided in diverse geographic settings, including urban (*n* = 42, 36.8%), suburban (*n* = 33, 28.9%), and rural (*n* = 30, 26.3%) communities. Religious service attendance varied, with 26.3% (*n* = 30) reporting never attending and 14.0% (*n* = 16) attending at least monthly. Professionally, the largest group consisted of recreational therapists (*n* = 35, 30.7%), followed by social workers (*n* = 22, 19.3%), special education teachers (*n* = 11, 9.6%), behavior specialists (*n* = 10, 8.8%), counselors (*n* = 9, 7.9%), and healthcare providers (*n* = 8, 7.0%). In terms of education, the largest group held a bachelor’s degree (*n* = 45, 39.5%), followed by the group with a master’s degree (*n* = 44, 38.6%). The majority of participants reported receiving professional training related to working with individuals with IDD (*n* = 93, 81.6%) and just over half had received training specific to working with LGBTQ+ individuals (*n* = 61, 53.5%). Participants’ experience working with individuals with IDD varied, with 43% (*n* = 49) reporting more than 10 years of experience. Additionally, 67.5% (*n* = 77) indicated prior experience working with an LGBTQ+ individual(s) with IDD.

**Table 1:** Participants’ sociodemographic characteristics.

Total	*n* (%) 114 (100)
Age	
18–24 years old	25 (21.9)
25–34 years old	34 (29.8)
35–44 years old	31 (27.2)
45–54 years old	16 (14.0)
55+ years old	8 (7.0)
Race/ethnicity	
White	104 (91.2)
Spanish, Hispanic, or Latino origin	5 (4.4)
Black or African American	4 (3.5)
Asian	3 (2.6)
Native Hawaiian or Other Pacific Islander	2 (1.8)
Other	4 (3.5)
Total	117 (102.6)
Gender identity	
Woman	96 (84.2)
Man	13 (11.4)
Gender nonbinary	4 (3.5)
Other	1 (0.9)
Sexual orientation identity	
Heterosexual	92 (80.7)
Bisexual/Pansexual	11 (9.6)
Lesbian	4 (3.5)
Queer	4 (3.5)
Gay	2 (1.8)
Other	1 (0.9)
Location	
In a city	42 (36.8)
In another metropolitan or suburban area	33 (28.9)
In a small or rural area	30 (26.3)
Missing	9 (7.9)
Religious services attendance	
Never	30 (26.3)
Less than once a year	16 (14.0)
At least yearly	17 (14.9)
At least monthly	16 (14.0)
At least weekly	25 (21.9)
Missing	10 (8.8)
Profession	
Recreational therapist	35 (30.7)
Social worker	22 (19.3)
Special education teacher	11 (9.6)
Behavior specialist	10 (8.8)
Counselor	9 (7.9)
Healthcare provider	8 (7.0)
K-12 teacher	6 (5.3)
Occupational therapist	6 (5.3)
Direct support provider	3 (2.6)
Program director	3 (2.6)
Psychologist	3 (2.6)
Dietitian	2 (1.8)
Nurse	2 (1.8)
Sexuality professional	2 (1.8)
Speech-language therapist	2 (1.8)
Other	12 (10.5)
Education	
Some college/associate/technical degree	14 (12.3)
Bachelor’s degree	45 (39.5)
Master’s degree	44 (38.6)
Professional and/or doctoral degree	11 (9.6)
Currently a student	
No	79 (69.3)
Yes	35 (30.7)
Received IDD-specific training	
Yes	93 (81.6)
No	19 (16.7)
Missing	2 (1.8)
Received LGBTQ+-specific training	
Yes	61 (53.5)
No	51 (44.7)
Missing	2 (1.8)
Years of experience working with population with IDD	
Less than 2 years	29 (25.4)
Between 2 and 10 years	34 (29.8)
More than 10 years	49 (43.0)
Missing	2 (1.8)
Prior experience working with LGBTQ+ individual(s) with IDD	
Yes	77 (67.5)
No	6 (5.3)
I don’t know	29 (25.4)
Missing	2 (1.8)

### Self-efficacy in LGBTQ+-affirmative service providing for individuals with IDD

Descriptive statistics for self-efficacy scores are presented in **Table 2**. On the Knowledge Application subscale (maximum possible score = 78) participants had a mean score of 42.50 (*SD* = 17.78, *Mdn* = 42.00, range: 13-78). The Awareness subscale (maximum possible score = 30) produced a mean score of 22.19 (*SD* = 5.85, *Mdn* = 23.00, range: 5-30). For Advocacy Skills (maximum possible score = 42), the mean score was 24.28 (*SD* = 10.05, *Mdn* = 25.00, range: 7-42). The Assessment subscale (maximum possible score = 24) had a mean score of 13.15 (*SD* = 5.74, *Mdn* = 13.00, range: 4-24). Participants reported the highest relative scores on the Relationship subscale (maximum possible score = 18), with a mean of 13.08 (*SD* = 4.22, *Mdn* =,14.50 range: 3-18) out of a maximum possible score of 18. The total self-efficacy score (maximum possible score = 192) had a mean of 115.15 (*SD* = 38.23, *Mdn* = 112.00, range: 32-192).

**Table 2:** Self-efficacy in LGBTQ+-affirmative service providing for individuals with IDD - descriptive statistics.

LGB-CSI			Knowledge application	Awareness	Advocacy skills	Assessment	Relationship	Total score
N	Valid		103	104	101	102	102	101
	Missing		11	10	13	12	12	13
Mean			42.50	22.19	24.28	13.15	13.08	115.15
Std. Error of Mean			1.75	.57	1.00	.57	.42	3.80
Median			42.00	23.00	25.00	13.00	14.50	112.00
Std. Deviation			17.78	5.85	10.05	5.74	4.22	38.23
Minimum			13	5	7	4	3	32
Maximum			78	30	42	24	18	192
Percentiles		25	30.00	19.00	17.00	9.00	10.00	89.50
		50	42.00	23.00	25.00	13.00	14.50	112.00
		75	55.00	26.00	33.50	18.00	16.00	143.50

**Figure 1:**
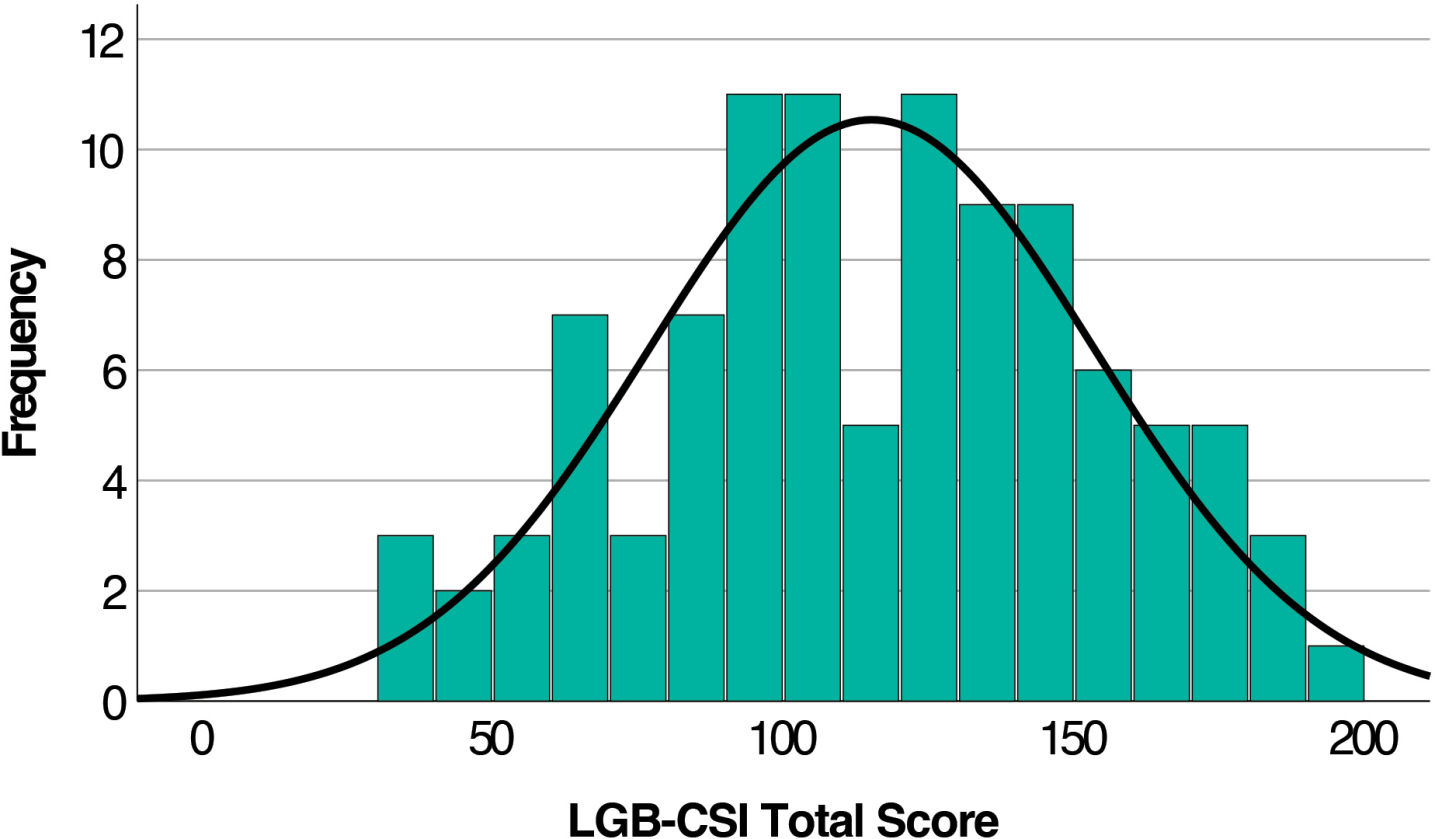
Data distribution for the LGB-CSI total score.

Distributional analyses indicated that scores across most subscales were approximately symmetrically distributed. However, the Awareness and Relationship subscales were negatively skewed, indicating that more participants scored toward the higher end of these subscales, with fewer participants reporting lower scores. Additionally, the total self-efficacy score and subscale distributions were relatively light-tailed compared to a normal distribution, indicating fewer extreme values than would be expected under normality (see **Table 3**, **Figure 1**).

**Table 3:** Self-efficacy in LGBTQ+-affirmative service providing for individuals with IDD - skewness and kurtosis.

LGB-CSI	Skewness		Kurtosis	
	Statistic	Std. error	Statistic	Std. error
Knowledge application	.05	.24	-.81	.47
Awareness	-.89	.24	.65	.47
Advocacy skills	-.08	.24	-.94	.48
Assessment	.01	.24	-.98	.47
Relationship	-.77	.24	-.21	.47
Total Score	-.12	.24	-.58	.48

### The LGBTQ+ knowledge and attitudes

Descriptive statistics for the LGBTQ+ Knowledge and Attitudes subscales are presented in **Table 4**. The mean score on the Hate subscale was 1.18 (*SD* = 0.36, *Mdn* = 1.00, range: 1.00-2.50). The Knowledge subscale had a mean of 2.57 (*SD* = 1.19, *Mdn* = 2.40, range: 1.00-5.80). The highest mean score was observed on the Civil Rights Attitudes subscale, with a mean of 5.43 (*SD* = 0.92, *Mdn* = 6.00, range: 2.40-6.00). The Religious Conflict subscale had a mean score of 2.01 (*SD* = 1.17, *Mdn* = 1.43, range: 1.00-4.71), and the Internalized Affirmativeness subscale demonstrated a mean of 3.95 (*SD* = 1.60, *Mdn* = 4.20, range: 1.00-6.00). Higher scores on each LGB-KAS subscale indicate greater endorsement of the construct measured by that subscale. Thus, the low mean score on the Hate subscale (*Mdn* = 1.18) indicates low endorsement of hostile, avoidant, or violent attitudes toward LGBTQ+ individuals in this sample. In contrast, the high mean score on the Civil Rights Attitudes subscale (*Mdn* = 5.43) indicates strong endorsement of LGBTQ+ civil rights.

**Table 4:** LGBTQ+ knowledge and attitudes - descriptive statistics.

LGBT-KAS		Hate	Knowledge	Rights	Religious conflict	Internalized affirmativeness
N	Valid	114	114	114	114	114
	Missing	0	0	0	0	0
Mean		1.18	2.57	5.43	2.01	3.95
Std. Error of Mean		.03	.11	.09	.11	.15
Median		1.00	2.40	6.00	1.43	4.20
Std. Deviation		.36	1.19	.92	1.17	1.60
Minimum		1.00	1.00	2.40	1.00	1.00
Maximum		2.50	5.80	6.00	4.71	6.00
Percentiles	25	1.00	1.60	5.00	1.00	2.40
	50	1.00	2.40	6.00	1.43	4.20
	75	1.17	3.40	6.00	3.00	5.25

**Figure 2:**
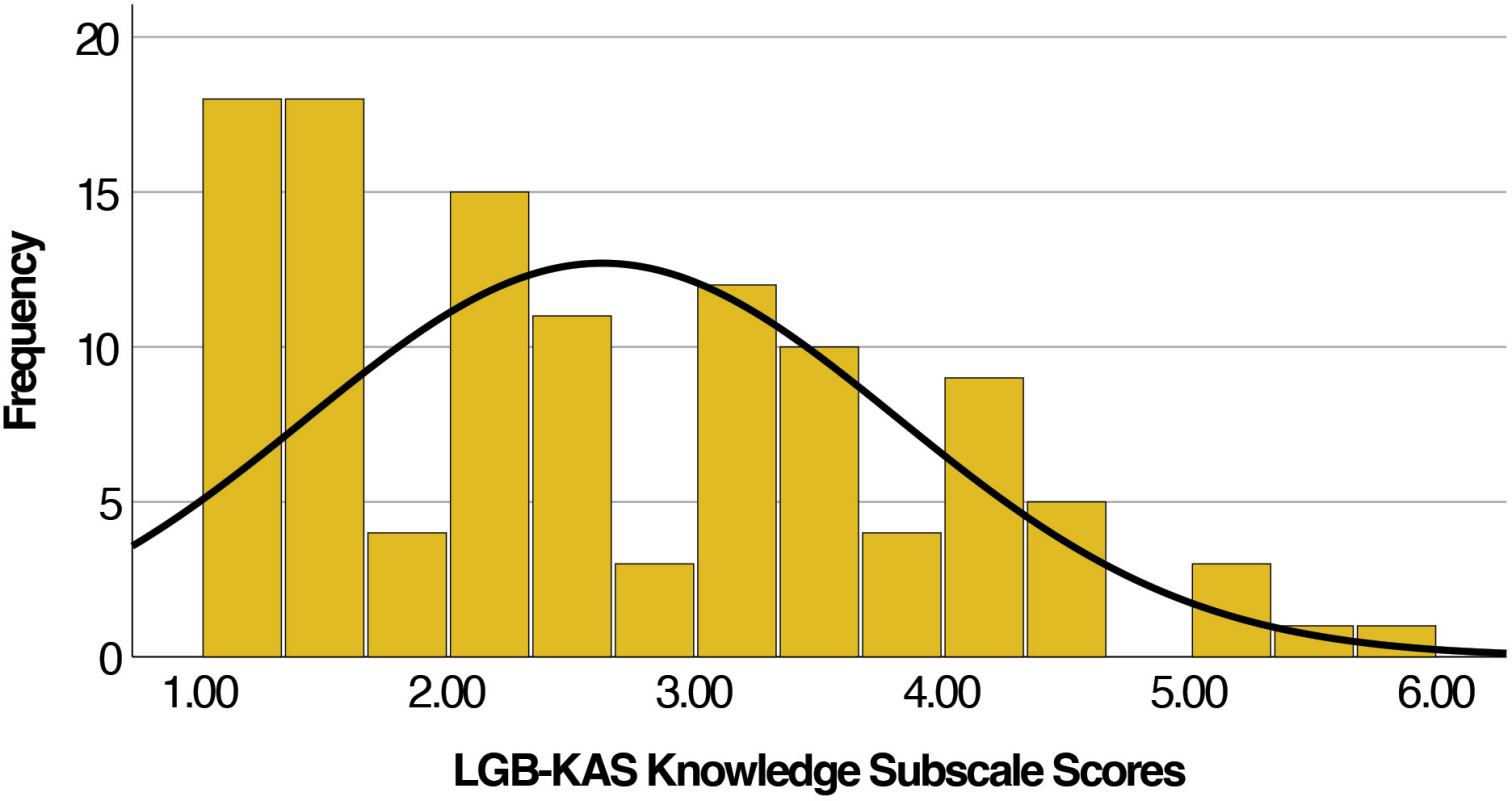
Data distribution for the LGB-KAS knowledge subscale.

Distributional analyses indicated that scores on the Internalized Affirmativeness subscale were approximately symmetric. In contrast, the Hate, Knowledge and Religious Conflict subscales were positively skewed indicating that more participants scored toward the lower end of these subscales, with fewer participants reporting higher scores, whereas the Civil Rights Attitudes subscale showed substantial negative skewness indicating that more participants scored toward the higher end of these subscales, with fewer participants reporting lower scores. The Hate subscale demonstrated a relatively heavy-tailed distribution, while the remaining subscales were indicative of a light-tailed distribution relative to a normal distribution (see **Table 5**, **Figure 2**).

**Table 5:** LGBTQ+ Knowledge and attitudes - skewness and kurtosis.

LGBT-KAS	Skewness		Kurtosis	
	Statistic	Std. error	Statistic	Std. error
Hate	2.17	.23	3.52	.45
Knowledge	.56	.23	-.57	.45
Civil Rights	-1.63	.23	1.65	.45
Religious Conflict	.90	.23	-.60	.45
Internalized Affirmativeness	-.33	.23	-1.22	.45

### Correlation between LGB-KAS and LGB-CSI

**Figure 3:**
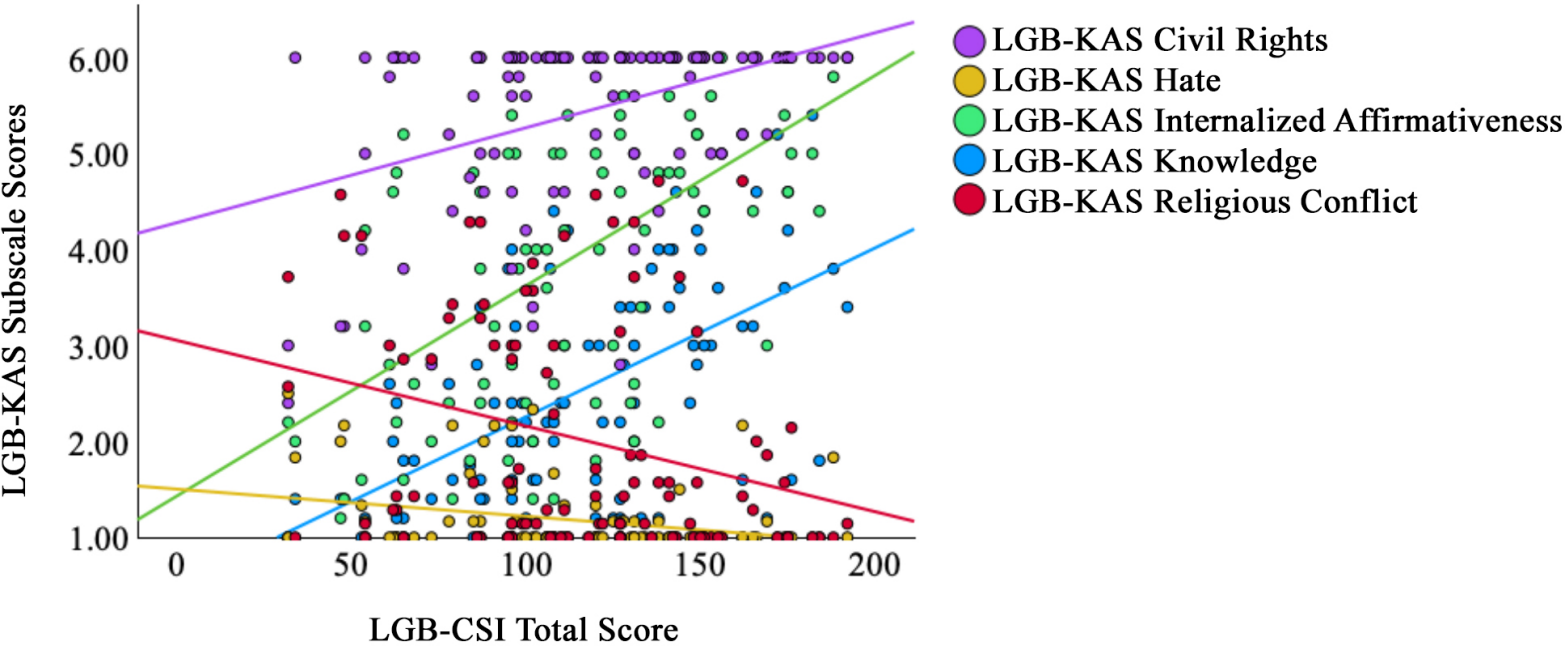
Correlation of LGB-CSI total score with LGB-KAS subscales.

Kendall’s tau correlations indicated significant associations between most LGB-KAS subscales and both the LGB-CSI total score and its subscales (see **Table 6**, **Figure 3**). The LGB-CSI total score demonstrated moderate positive correlations with LGB-KAS Knowledge (τ = .424, *p* <.001), Internalized Affirmativeness (τ = .372, *p* <.001), and Civil Rights Attitudes (τ = .276, *p* <.001). Significant negative correlations were observed with the Hate (τ=-.235, p=.003) and Religious Conflict (τ = -.203, *p* = .004) subscales. These correlations indicate that participants with higher LGBTQ+ knowledge, stronger internalized affirmativeness, and greater support for LGBTQ+ civil rights tended to report higher self-efficacy in LGBTQ+-affirmative practice. In contrast, participants with higher scores on Hate and Religious Conflict tended to report lower self-efficacy.

**Table 6:** Correlation of self-efficacy in LGBTQ+-affirmative service providing for individuals with IDD (LGB-SCI) and LGBTQ+ knowledge and attitudes (LGB-KAS).

LGB-CSI	LGB-KAS, Kendall’s correlation τ (p-value)				
	Hate	Knowledge	Civil Rights	Religious conflict	Internalized affirmativeness
Knowledge application	**-.203 (.009)**	**.472 (<.001)**	**.288 (<.001)**	**-.216 (.002)**	**.389 (<.001)**
Awareness	**-.208 (.008)**	**.310 (<.001)**	**.259 (<.001)**	**-.203 (.004)**	**.392 (<.001)**
Advocacy skills	**-.224 (.004)**	**.309 (<.001)**	**.201 (.008)**	**-.181 (.011)**	**.264 (<.001)**
Assessment	-.095 (.230)	**.224 (.001)**	.132 (.085)	-0.28 (.698)	**.188 (.008)**
Relationship	**-.259 (.001)**	.**328 (<.001)**	**.288 (<.001)**	**-.218 (.003)**	**.380 (<.001)**
Total score	**-.235 (.003)**	**.424 (<.001)**	**.276 (<.001)**	**-.203 (.004)**	**.372 (<.001)**

Bold values indicate statistically significant results at p < .05.

Across LGB-CSI subscales, a consistent pattern emerged. Knowledge Application demonstrated the strongest positive associations with LGBTQ+ Knowledge (τ = .472, *p* <.001) and Internalized Affirmativeness (τ = .389, *p* <.001) and weaker negative associations with Hate (τ = -.203, *p* = .009) and Religious Conflict (τ = -.216, *p* = .002). Awareness and Advocacy Skills showed similar moderate positive correlations with Knowledge and Internalized Affirmativeness (τs = .264–.392, all *ps* <.01) and weaker negative correlations with Hate and Religious Conflict (τs = −.181 to −.224, all *ps* ≤.011). Relationship demonstrated strong positive correlations with Knowledge (τ = .328, *p* <.001) and Internalized Affirmativeness (τ = .380, *p* <.001) and negative correlations with the Hate (τ = -.259, *p* = .001) and Religious Conflict (τ = -.218, *p* = .003). Although somewhat weaker, Assessment subscale followed the same directional trends.

Overall, higher levels of LGBTQ+ knowledge, affirming attitudes, and support for civil rights were associated with greater self-efficacy in providing LGBTQ+-affirmative services. In contrast, higher levels of hate and religious conflict were associated with lower self-efficacy.

### Associations between LGB-CSI and sociodemographic covariates

Significant differences in LGB-SCI total and subscale scores were observed across several sociodemographic variables (see **Table 7**). Age was significantly associated with the Awareness (*F* = 4.299, *p* = .003) and Relationship (*F* = 2.501, *p* = .047) subscales, indicating variability across age groups. Mean scores were generally higher among older age groups, suggesting greater self-efficacy in these domains among older participants. Participants’ LGBTQ+ identity was significantly associated with the LGB-CSI total score and all subscales, with individuals identifying as LGBTQ+ demonstrating higher self-efficacy compared to non-LGBTQ+ participants.

**Table 7:** Associations between self-efficacy in LGBTQ+-affirmative service providing for individuals with IDD and covariates.

Covariates	F (p-value)					
	Knowledge application	Awareness	Advocacy skills	Assessment	Relationship	Total score
Age	2.170(.078)	**4.299(.003)**	2.208(.074)	.872(.484)	**2.501(.047)**	2.165(.079)
LGBTQ+ identity	**17.960 (<.001)**	**9.960(.002)**	**5.865(.017)**	**5.581(.020)**	**14.682 (<.001)**	**14.734 (<.001)**
Location	1.768(.176)	2.321(.103)	1.392(.254)	.164(.849)	1.040(.357)	1.388(.254)
Religious services attendance	**2.520(.046)**	**3.314(.014)**	1.865(.123)	.781(.540)	**3.685(.008)**	**2.634(.039)**
Education level	2.194(.094)	**3.312(.023)**	1.861(.141)	.227(.877)	1.378(.254)	2.107(.104)
Student status	2.153(.145)	**5.735(.018)**	2.266(.135)	.191(.663)	2.061(.154)	2.342(.129)
Training – IDD	2.582(.111)	1.024(.314)	2.075(.153)	.911(.342)	2.426(.122)	1.707(.194)
Training - LGBTQ+	**15.802 (<.001)**	3.114(.081)	2.112(.149)	2.206(.141)	2.714(.103)	**7.911(.006)**
Years of experience working with individuals with IDD	1.351(.264)	2.904(.059)	2.864(.062)	.258(.773)	1.188(.309)	1.757(.178)
Prior experience working with LGBTQ+ individual(s) with IDD	**10.097 (<.001)**	**7.732 (<.001)**	**10.221 (<.001)**	3.619(0.30)	**6.594(.002)**	**11.161 (<.001)**

Bold values indicate statistically significant results at p < .05.

Religious service attendance was significantly associated with the LGB-CSI total score and the Knowledge Application, Awareness, and Relationship subscales. Participants attending religious services at least weekly demonstrated the lowest mean scores across following domains: total score (*X¯* = 94.40, *SD* = 43.25), Knowledge Application (*X¯* = 33.12, *SD* = 19.94), Awareness (*X¯* = 18.84, *SD* = 6.83), and Relationship (*X¯* = 10.52, *SD* = 5.05) subscale score. The Advocacy Skills and Assessment were not significantly associated with religious attendance. Overall, these findings suggest that more frequent religious service attendance was associated with lower overall self-efficacy and lower self-efficacy in Knowledge Application, Awareness, and Relationship domains.

Education level was generally not associated with self-efficacy, except for the Awareness subscale. Participants holding a master’s degree reported the highest Awareness scores (*X¯* = 23.78, *SD* = 5.11), whereas those with some college, an Associate’s, or a technical degree reported the lowest (*X¯* = 18.15, *SD* = 7.22). Awareness scores were also significantly associated with participants’ student status, with non-students having higher scores (*X¯* = 23.13, *SD* = 5.21) than students (*X¯* = 20.26, *SD* = 6.66).

Receiving training for working with individuals with IDD was not significantly associated with LGB-CSI total or subscale scores. In contrast, receiving training specific to working with the LGBTQ+ population was significantly associated with higher total self-efficacy and Knowledge Application scores. Participants who had received LGBTQ+ training reported higher total scores (*X¯* = 123.64, *SD* = 36.07) than those who had not (*X¯* = 103.07, *SD* = 36.77). Similarly, Knowledge application scores were significantly higher among those who received LGBTQ+-specific training (*X¯* = 47.98, *SD* = 16.23) compared to those who did not (*X¯* = 35.04, *SD* = 16.51).

Years of professional experience working with individuals with IDD were not significantly associated with self-efficacy scores. However, prior experience working with LGBTQ+ individual(s) with IDD was significantly associated with higher LGB-CSI total scores and higher Knowledge Application, Awareness, Advocacy Skills, and Relationship subscale scores, compared to those without such experience or those unsure whether they have ever worked with LGBTQ+ individual(s) with IDD.

### Hierarchical regression analysis of factors associated with self-efficacy

A hierarchical multiple regression analysis was conducted to examine predictors of self-efficacy in providing LGBTQ+-affirmative service to LGBTQ+ individuals with IDD (LGB-CSI Total Score). Predictors included the following variables: LGB-KAS Internalized Affirmativeness, LGB-KAS Knowledge, and Experience working with LGBTQ+ individual(s) with IDD. Correlation analysis indicated that all predictors were positively associated with the LGB-CSI total score, with the strongest relationships observed between self-efficacy and LGB-KAS Knowledge, and between LGB-KAS Knowledge and Internalized Affirmativeness (See **Table 8**).

**Table 8:** Regression model for the self-efficacy in LGBTQ+-affirmative service providing for individuals with IDD (LGB-SCI) - Pearson correlation.

Variable	LGB-CSI Total score	LGB-KAS Internalized affirmativeness	LGB-KAS Knowledge
LGB-KAS Internalized affirmativeness	.509		
LGB-KAS Knowledge	.576	.686	
Prior experience working with LGBTQ+ individual(s) with IDD	.432	.412	.367

In Model 1, Internalized Affirmativeness was entered as the sole predictor and accounted for 25.1% of the variance in self-efficacy (adjusted *R²* = .251), indicating a significant positive association.

Model 2 added LGBTQ+ Knowledge, which explained an additional 9.2% of the variance (adjusted ΔR² = .092), increasing the total explained variance to 34.3% (adjusted *R²* = .343). In this model, LGBTQ+ Knowledge emerged as a strong positive predictor of self-efficacy.

Model 3 incorporated prior experience working with LGBTQ+ individual(s) with IDD, explaining an additional 3.7% in the regression model for increasing self-efficacy of variance (adjusted Δ*R²* = .037) and increasing the total explained variance to 38.0% (adjusted *R²* = .380). In the final model, LGBTQ+ Knowledge remained a significant positive predictor, and prior experience working with LGBTQ+ individual(s) with IDD also contributed significantly. Internalized Affirmativeness, although significant in the initial model, was no longer a significant predictor after the inclusion of Knowledge and LGBTQ+ Experience (see **Tables 9**, **10**).

**Table 9:** Regression model for the self-efficacy in LGBTQ+-affirmative service providing for individuals with IDD - test statistics.

Model	Explanatory variable(s)	*F*	*df*	*p*-value	*R^2^* adjusted
1	LGB-KAS Internalized affirmativeness	34.234	1	**<0.01**	.251
2	LGB-KAS Internalized affirmativeness, LGBT-KAS Knowledge	14.626	1	**<0.01**	.343
3	LGBT-KAS Internalized affirmativeness, LGBT-KAS Knowledge, Prior experience working with LGBTQ+ individual(s) with IDD	6.785	1	**.011**	.380

Dependent Variable: LGB-CSI Total Score.

Bold values indicate statistically significant results at p < .05.

**Table 10:** Regression model for the self-efficacy in LGBTQ+-affirmative service providing for individuals with IDD – regression coefficients.

Explanatory variable(s)	Coefficients	95% *CI*	*p*-value
Constant	45.949	26.66, 65.24	**<.001**
LGBT-KAS Internalized affirmativeness	3.426	-1.77, 8.62	.194
LGBT-KAS Knowledge	12.60	5.60, 19.60	**<.001**
Prior experience working with LGBTQ+ individual(s) with IDD	9.662	2.30, 17.03	**.011**

CI, confidence interval.

Dependent variable: LGB-CSI Total score.

Bold values indicate statistically significant results at p < .05.

Diagnostic statistics indicated that regression assumptions were met. The Durbin-Watson statistic (2.034) suggested no evidence of autocorrelation in the residuals, and multicollinearity diagnostics (tolerance >.50; VIF < 2.0) indicated sufficient independence among predictors. Overall, LGBTQ+ Knowledge emerged as the most robust predictor of self-efficacy, with additional contributions from direct experience working with LGBTQ+ individuals with IDD.

## Discussion

This study identified LGBTQ+-specific knowledge and direct experiential exposure as the strongest correlates of self-efficacy in LGBTQ+-affirmative practice among DSPs working with individuals with IDD. Grounded in Bandura’s self-efficacy theory (35, 36), the findings suggest that specific knowledge, LGBTQ+-specific professional exposure and experience, and affirming attitudes are associated with DSPs’ confidence to provide affirming services at the intersection of disability and sexual and gender minority identities to LGBTQ+ individuals with IDD. By centering the intersection of disability, sexual orientation, and gender identity, this research contributes to a nuanced understanding of the competencies that support LGBTQ+-affirmative practice across diverse professional settings.

Consistent with self-efficacy theory, LGBTQ+ knowledge emerged as the main factor associated with self-efficacy. Although the Knowledge subscale was the strongest statistical correlate of self-efficacy in the regression model, this finding should be interpreted cautiously. While the subscale is intended to assess foundational knowledge of LGBTQ+ identities, communities, and relevant historical and sociocultural contexts, higher scores may also reflect greater exposure to LGBTQ+ communities, affirming social contexts, or related professional experiences. Therefore, knowledge should not be interpreted as a purely cognitive construct or as causally producing higher self-efficacy. Rather, in this sample, LGBTQ+-related knowledge was strongly associated with perceived self-efficacy and may represent a combination of information, exposure, and affirming attitudes.

In hierarchical regression models, LGBTQ+ knowledge accounted for a significant portion of variance in self-efficacy beyond internalized affirmativeness alone, underscoring its central role in developing professional confidence. This finding aligns with Bandura’s (35) conceptualization of mastery experiences as a foundational source of self-efficacy, developed through accurate information and successful performance in relevant tasks. These findings are consistent with prior research demonstrating that LGBTQ+-specific knowledge is positively associated with affirmative competence and self-efficacy among mental health professionals, psychology trainees, and educators (68–70). Across these professional contexts, structured training programs that increased knowledge of LGBTQ+ identities and issues were associated with increase in perceived competence. The present study extends this literature by demonstrating similar associations within professional practices serving individuals with IDD and by explicitly examining self-efficacy in the context of the intersection of LGBTQ+ and IDD identities.

Internalized affirmativeness, reflecting proactive engagement with LGBTQ+ issues and comfort with sexual orientation and gender diversity, was positively associated with self-efficacy when considered independently, but its association weakened when knowledge and experience were included. These findings underscore that affirming values alone may be insufficient without targeted knowledge and skill development. Similar distinctions have been observed in educational settings, where general teaching self-efficacy was not associated with self-efficacy for teaching LGBTQ+ students. Instead, heterosexist beliefs (i.e., beliefs or assumptions that privilege heterosexuality as normal or preferred and stigmatize LGBTQ+ identities) were associated with lower domain-specific confidence in teaching LGBTQ+ students (71). These findings highlight the importance of integrating both cognitive and affective dimensions in professional development initiatives aiming to prepare practitioners to serve all individuals with IDD.

Negative attitudes, including higher scores on the Hate and Religious Conflict subscales, were consistently associated with lower self-efficacy. In this context, Hate subscale scores reflect hostile, avoidant, or violent attitudes toward LGBTQ+ individuals, whereas Religious Conflict subscale scores reflects conflict between religious beliefs and acceptance of LGBTQ+ identities. From a theoretical perspective, these associations may reflect the influence of emotional and physiological states on self-efficacy beliefs (35), where discomfort, avoidance, or internal conflict regarding LGBTQ+ identities may decrease willingness to engage affirmatively with clients, patients, or students. Conversely, higher Civil Rights Attitudes subscale scores reflecting greater support for LGBTQ+ civil rights such as marriage equality, parenting rights, healthcare access, and recognition of same-sex partners in healthcare decision-making were positively associated with higher self-efficacy, indicating that broader sociopolitical values may reinforce professional confidence in LGBTQ+-affirming practice.

Findings related to religious service attendance and religious conflict should be interpreted with caution. The observed associations do not indicate that religiosity itself is incompatible with LGBTQ+-affirmative practice. Rather, within this sample, more frequent religious service attendance and higher religious conflict scores were associated with lower self-efficacy in some domains. Future research should examine how religious identity, institutional context, cultural background, and affirming religious frameworks may shape professionals’ attitudes and confidence in LGBTQ+-affirmative practice.

Importantly, prior professional experience with LGBTQ+ individuals with IDD—but not general professional experience with individuals with IDD—was associated with higher self-efficacy. Similarly, general IDD training was not associated with self-efficacy for LGBTQ+-affirmative practice, highlighting the limitations of broad disability training when it lacks targeted content addressing sexual orientation and gender identity. This finding suggests that intersectionally- relevant exposure may be particularly important for building confidence in LGBTQ+-affirmative care and services. Moreover, these findings support previous research demonstrating that self-efficacy is context-specific and strengthened through experiences closely linked to the domain of interest (53–57, 72, 73). Studies with psychology and counselor trainees similarly show that supervised experience working directly with LGBTQ+ individuals is associated with higher LGBTQ+-affirmative self-efficacy (68, 69). The present study extends these contexts by suggesting that experiential exposure must be intersectionally- relevant, specifically involving LGBTQ+ individuals with IDD, rather than limited to general disability-related experience.

At the same time, these findings should not be interpreted as indicating that general disability-related training or experience is unimportant. Rather, general IDD training and professional experience may provide an essential foundation for working with individuals with IDD, but they may be insufficient on their own for developing self-efficacy in LGBTQ+-affirmative practice unless they explicitly address sexual orientation, gender identity, and affirming service provision. Because most participants reported IDD-related training and experience, these factors may also be difficult to fully separate from other professional preparation and practice experiences.

Demographic findings suggest that self-efficacy may develop cumulatively across the professional lifespan. Age was significantly associated with awareness and relational self-efficacy, with older DSPs reporting higher scores—potentially reflecting accumulated mastery experiences and professional exposure. Educational level was generally not associated with self-efficacy, except for awareness, suggesting potential gaps in pre-service preparation. These findings are aligned with prior studies showing that self-efficacy may develop through increasing experiences over the professional lifespan when initial training environments may insufficiently prepare emerging professionals (74–77), highlighting the need for continuing professional development opportunities.

Consistent with prior evaluations of LGBTQ+-affirmative training programs (68, 70, 78–83), educational interventions framed within the self-efficacy theory appear critical for strengthening knowledge, reducing bias, and enhancing perceived competence across professional settings. In alignment with Bandura’s framework (35), training may be most effective when it incorporates structured mastery experiences (e.g., supervised practice with LGBTQ+ individuals with IDD), vicarious learning through modeling LGBTQ+-affirmative practices, affirming feedback and mentorship from knowledgeable supervisors, and strategies to address emotional discomfort or bias. Programs that simultaneously build domain-specific knowledge and affirming attitudes while challenging cis- and heteronormative assumptions may be particularly effective (70, 78, 83–88).

Prior LGBTQ+-affirmative training programs have increased professional knowledge by providing structured content on LGBTQ+ terminology, identity development, minority stress, stigma and discrimination, affirming communication, and strategies for creating inclusive service environments (68, 70, 78, 79). In healthcare and clinical settings, trainings focused on transgender-affirming care, inclusive policies, and culturally responsive provider behaviors have also been associated with improvements in knowledge, attitudes, self-efficacy, and/or intentions (80–83). These knowledge-building components may strengthen self-efficacy by linking conceptual understanding with concrete affirming practice skills.

While prior research has examined self-efficacy in LGBTQ+-affirmative counseling, education, and healthcare contexts, few studies have explicitly examined affirmative practice within disability services, and none to our knowledge have centered the intersection of LGBTQ+ and IDD identities. By situating self-efficacy within an intersectional disability framework, this study broadens the scope of affirmative practice research and highlights the importance of domain-specific competence in addressing compounded marginalization.

From an intersectional perspective, LGBTQ+ individuals with IDD navigate overlapping systems of ableism, heteronormativity and cisnormativity that shape their access to inclusive and affirming care (2, 4). Within these broader structural contexts, provider self-efficacy represents an essential factor that may influence whether inequities are reinforced or mitigated within diverse service-providing settings. Confidence in addressing sexual orientation and gender identity among individuals with IDD is not merely a matter of individual competence, but a critical component of equitable person-centered service delivery. Higher self-efficacy may support DSPs to advocate for inclusive policies, address structural barriers to holistic care, and create and normalize environments in which LGBTQ+ individuals with IDD are respected, visible, and supported in their identities, relationships, and overall well-being.

The adaptation of the LGB-CSI represents an important limitation. Although the instrument was conceptually appropriate for assessing LGBTQ+-affirmative practice self-efficacy, its original validation did not focus on professionals working with LGBTQ+ individuals with IDD. Modifying items to refer to LGBTQ+ individuals with IDD introduced a distinct professional and social context that may affect how respondents interpreted the items. Self-efficacy in this context may involve additional competencies related to communication, autonomy, supported decision-making, dependency, and vulnerability to abuse. Although internal consistency estimates in the present sample provided preliminary evidence of reliability, these findings do not constitute full validation of the adapted measure. Future research should examine the factor structure, construct validity, and measurement invariance of the adapted LGB-CSI among professionals working with individuals with IDD.

Recruitment procedures may have introduced selection bias. The sample shown limited diversity with predominantly white participants majorly identifying as women, therefore limiting generalizability to more diverse DSP populations. Participants were recruited using non-probability methods, including through professional listservs, professional and scientific associations, public provider directories, Facebook groups, and snowball sampling. These recruitment strategies may have been more likely to reach professionals who were connected to professional networks, active in online communities, or already interested in LGBTQ+-affirmative practice or IDD-related services. Consequently, DSPs who were less connected to such networks, less active online, or less comfortable engaging with LGBTQ+-related topics may have been less likely to participate. These factors limit the generalizability of the findings.

The study’s cross-sectional design limits causal inference, and reliance on self-report measures may introduce social desirability bias. Although the final regression model accounted for 38% of the variance in self-efficacy, it focused on individual-level factors, including LGBTQ+-related knowledge, affirming attitudes, and prior experience. Additional factors, including organizational climate, institutional policies, leadership support, access to LGBTQ+-affirmative training, workplace norms, and regional sociopolitical context may influence whether DSPs feel prepared and supported to provide LGBTQ+-affirming services (83, 89, 90). Future research should examine these multi-level factors and evaluate how organizational contexts interact with individual-level knowledge, attitudes, and experience to shape LGBTQ+-affirmative practice.

This study advances theoretical and practical understanding of LGBTQ+-affirmative practice by identifying key factors associated with self-efficacy within the intersecting contexts of disability and sexual and gender minority identities. By emphasizing domain-specific knowledge, affirming attitudes, and intersectionally-relevant experience, and by framing these within a self-efficacy framework, these findings offer actionable insights for improving inclusive service provision and affirmative care for LGBTQ+ individuals with IDD. Integrating these elements into professional preparation and continuing education holds potential for improving health equity and advancing the well-being and quality of life of LGBTQ+ people with IDD.

## Data Availability

The datasets presented in this article are not readily available due to ethical and confidentiality considerations. Requests to access the datasets should be directed to isimics@iu.edu.
